# Optimal flickering light stimulation for entraining gamma rhythms in older adults

**DOI:** 10.1038/s41598-022-19464-2

**Published:** 2022-09-16

**Authors:** Yeseung Park, Kanghee Lee, Jaehyeok Park, Jong Bin Bae, Sang-Su Kim, Do-Won Kim, Se Joon Woo, Seunghyup Yoo, Ki Woong Kim

**Affiliations:** 1 Department of Neuropsychiatry, Seoul National University Bundang Hospital, Seongnam, Republic of Korea; 2 Department of Brain and Cognitive Science, Seoul National University College of Natural Sciences, Seoul, Republic of Korea; 3 School of Electrical Engineering, Korea Advanced Institute of Science and Technology (KAIST), Daejeon, Republic of Korea; 4 Department of Biomedical Engineering, Chonnam National University, Yeosu, Republic of Korea; 5 Department of Ophthalmology, Seoul National University College of Medicine, Seoul, Republic of Korea; 6 Department of Ophthalmology, Seoul National University Bundang Hospital, Seongnam, Republic of Korea; 7 Department of Psychiatry, Seoul National University College of Medicine, Seoul, Republic of Korea

**Keywords:** Neuroscience, Psychology, Health care

## Abstract

With aging, optimal parameters of flickering light stimulation (FLS) for gamma entrainment may change in the eyes and brain. We investigated the optimal FLS parameters for gamma entrainment in 35 cognitively normal old adults by comparing event-related synchronization (ERS) and spectral Granger causality (sGC) of entrained gamma rhythms between different luminance intensities, colors, and flickering frequencies of FLSs. ERS entrained by 700 cd/m^2^ FLS and 32 Hz or 34 Hz FLSs was stronger than that entrained by 400 cd/m^2^ at Pz (*p* < 0.01) and 38 Hz or 40 Hz FLSs, respectively, at both Pz (*p* < 0.05) and Fz (*p* < 0.01). Parieto-occipital-to-frontotemporal connectivities of gamma rhythm entrained by 700 cd/m^2^ FLS and 32 Hz or 34 Hz FLSs were also stronger than those entrained by 400 cd/m^2^ at Pz (*p* < 0.01) and 38 Hz or 40 Hz FLSs, respectively (*p* < 0.001). ERS and parieto-occipital-to-frontotemporal connectivities of entrained gamma rhythms did not show significant difference between white and red lights. Adverse effects were comparable between different parameters. In older adults, 700 cd/m^2^ FLS at 32 Hz or 34 Hz can entrain a strong gamma rhythm in the whole brain with tolerable adverse effects.

## Introduction

Gamma rhythms play prominent roles in object perception^1^, control of visual attention^2^, word encoding^3^, the maintenance of relevant items in working memory^4^, crossmodal semantic matching^5^, and short-term memory retention^6^. In patients with Alzheimer’s disease (AD), gamma rhythm response is delayed and its power is reduced compared with the cognitively healthy elderly^7–9^. Recently, gamma rhythms entrained by photic stimulation using external light flickering at 40 Hz were found to reduce beta amyloid (Aβ) and phosphorylated tau in the brains of transgenic AD mice and to improve their cognition^10–12^. These results suggest that gamma entrainment using flickering light stimulation (FLS) may be a promising non-invasive intervention for preventing AD or modifying the course of AD.

The efficiency of gamma entrainment is influenced by the luminance intensity, color (wavelength), and flickering frequency of FLS^13–19^. In our previous study, FLS with relatively high luminance (700 cd/m^2^ or 400 cd/m^2^) and longer wavelength (red or white) entrained stronger and more widely spread gamma rhythms than the FLS with weaker (100 cd/m^2^ or 10 cd/m^2^) and shorter wavelength (green or blue), respectively, in younger adults^19^. Furthermore, the lights flickering at 34–38 Hz entrained stronger and more widely spread gamma rhythms than those flickering at other frequencies, including 40 Hz^19^.

However, the optimal parameters of FLS for gamma entrainment in older adults may be different from those in younger adults. When exposed to the Rubin vase flickering at 36 Hz, gamma rhythms of 36 Hz were entrained in both younger and older adults, but their power in older adults was lower than that in younger adults^20^. When exposed to visual Cartesian gratings that had spatial gamma frequencies, the power and central frequency of entrained gamma rhythms in older adults were lower than those in younger adults^21^. These age-associated differences in gamma entrainment using photic stimulation may be attributable to the age-associated changes in the eyes and brain. With aging, retinal illuminance becomes less effective due to declined pupillary miosis and color discrimination. The lens-related reduction of illuminance is not equal for all wavelengths, and the majority of color defects in old people is blue-yellow type^22,23^. The center frequency of the gamma rhythms gradually decreases with advancing age^21^.

This study is aimed at finding optimal luminance intensity (400 cd/m^2^ versus 700 cd/m^2^), color (white and red), and flickering frequency (32 Hz, 34 Hz, 36 Hz, 38 Hz and 40 Hz) of FLS for entraining gamma rhythms in cognitively normal older adults by comparing the event-related synchronization (ERS) of gamma rhythms in the visual cortex and the spectral Granger causality (sGC) of gamma rhythm connectivity from visual cortex to other brain regions.

## Results

### Entrainment of gamma rhythm

**Figure 1:**
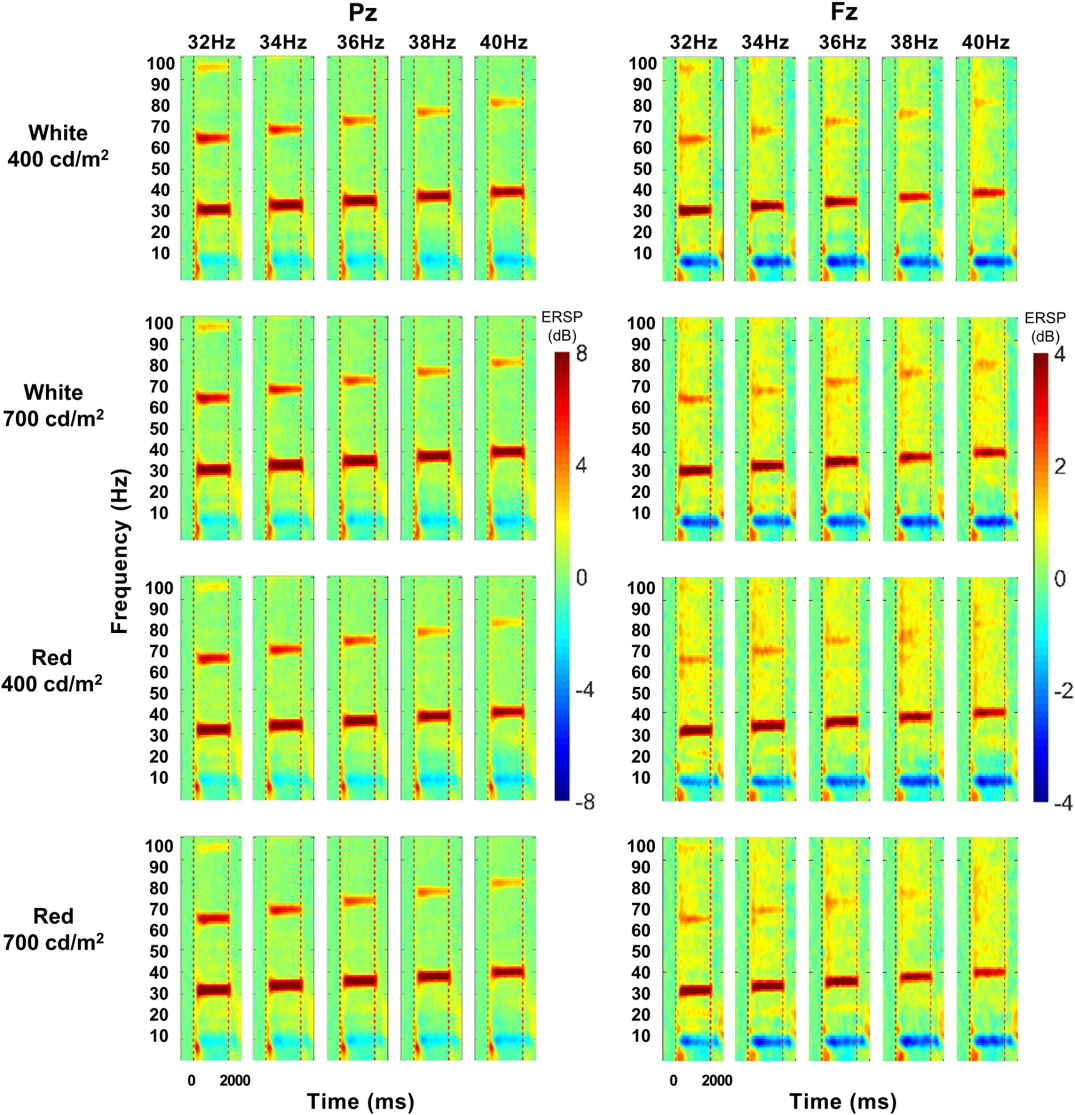
Comparison of grand-average event-related spectral perturbation (ERSP) of steady-state visually evoked potentials induced by flickering light stimulation (FLS) between colors, luminance intensities, and flickering frequencies. Each column shows ERSP from 750 ms before the onset of FLS to 750 ms after the offset of FLS.

**Figure 2:**
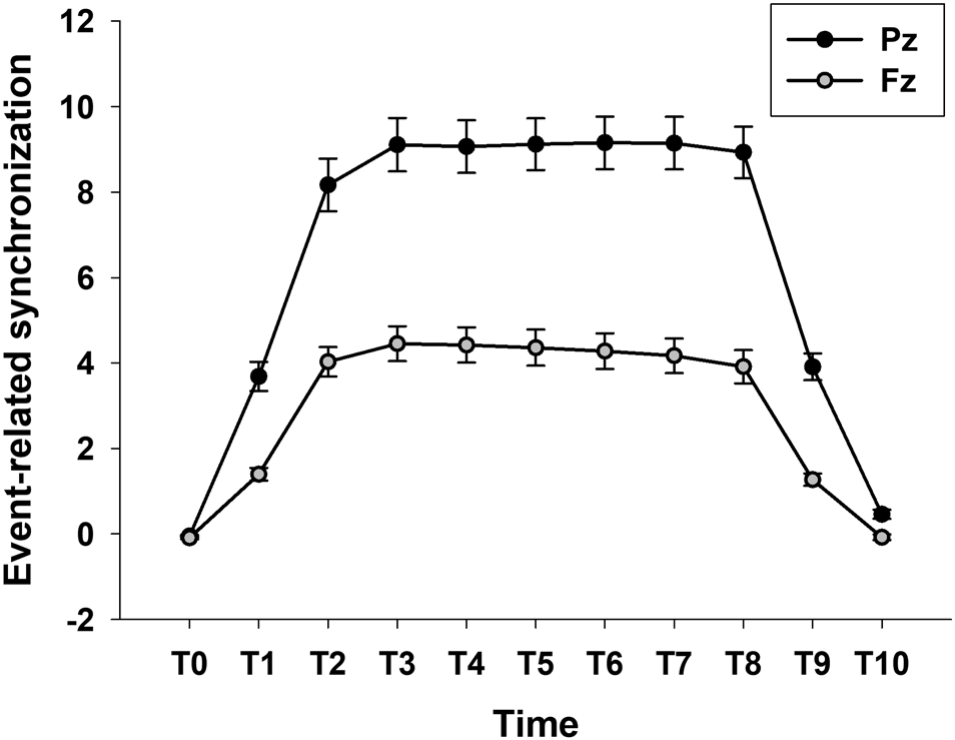
Main effect of time windows on grand-average event-related synchronization (ERS) of steady-state visually evoked potentials induced by flickering light stimulation (FLS). T0–T10 indicates 250 ms time windows from 250 ms before the onset of FLS to 500 ms after the offset of FLS.

As shown in Fig. 1, the spectral power of the steady-state visually evoked potential (SSVEP) starts to increase at the fundamental and harmonic frequencies of FLS after FLS onset, lasts during the FLS, and diminishes after FLS offset at both Pz and Fz. The averages of event-related desynchronization/event-related synchronization (ERD/ERS) in each time window after FLS onset were positive (ERS), indicating that the spectral power of SSVEP increased after FLS. The main effect of the time window on ERS was significant at both Pz and Fz (F_10, 340_ = 170.699, *p* < 0.001, ηp^2^ = 0.834 at Pz; F_10, 340_ = 96.205, *p* < 0.001, ηp^2^ = 0.739 at Fz, Fig. 2). At both Pz and Fz, ERS during FLS (T1–T9) was higher than ERS before FLS (T0) and that after FLS (T10).

### Effects of luminance intensity and color of FLS on the gamma entrainment

**Figure 3:**
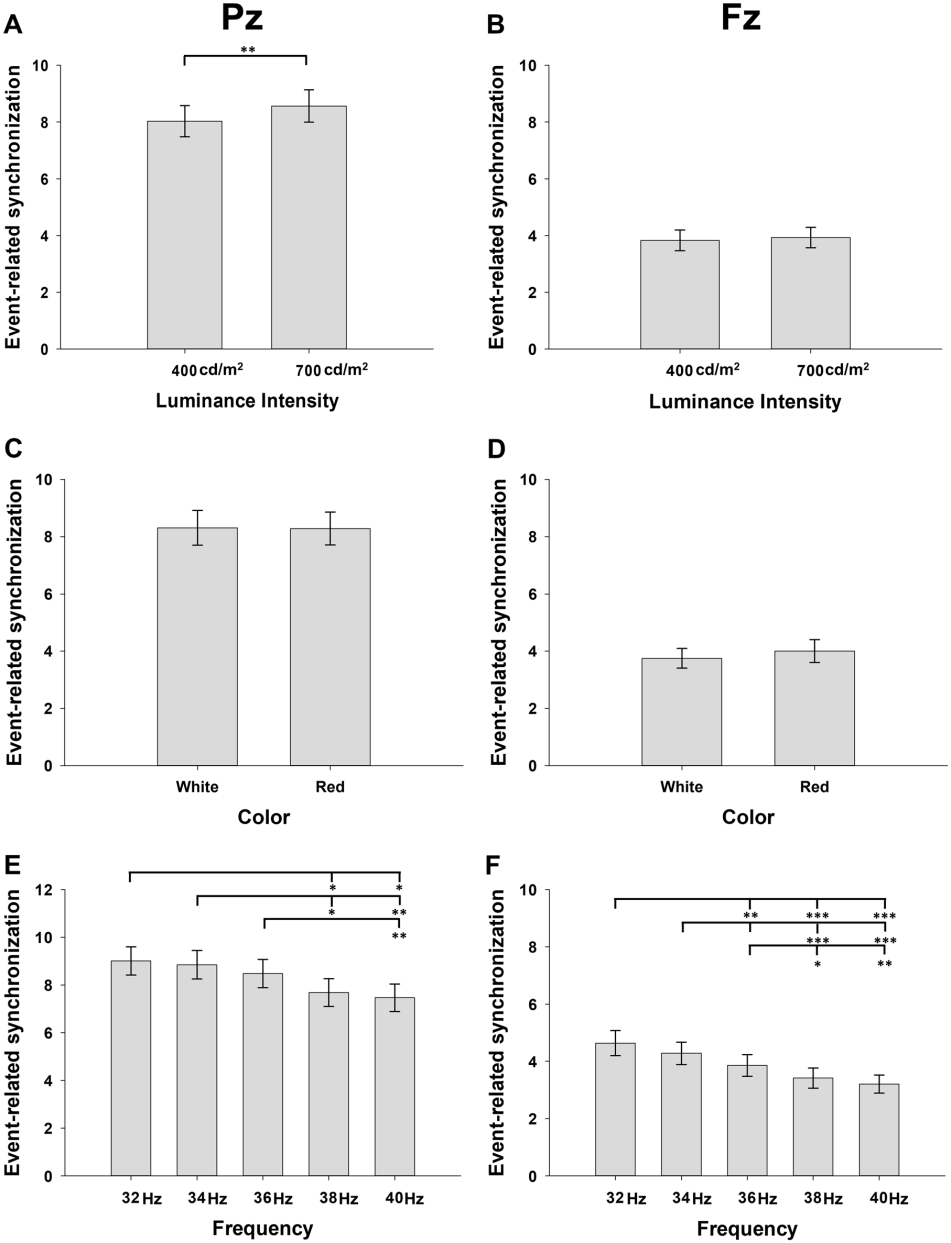
Main effects of the luminance intensities, colors, and flickering frequencies of flickering light stimulations on event-related synchronization (ERS) (**A**) and (**B**) demonstrate the main effect of luminance intensities, (**C**) and (**D**) demonstrate the main effect of colors, (**E**) and (**F**) demonstrate the main effect of flickering frequencies. Error bars indicate standard errors. ^*^p < 0.05, ^**^p < 0.01, and ^***^p < 0.001 by repeated-measures analysis of variance.

The main effect of the luminance intensity of FLS on ERS was significant at Pz (F_1, 34_ = 10.536, *p* = 0.003, ηp^2^ = 0.237, Fig. 3A) but not at Fz (*p* = 0.385, ηp^2^ = 0.022, Fig. 3B). At Pz, 700 cd/m^2^ FLS entrained stronger SSVEP than 400 cd/m^2^ FLS (*p* = 0.003). On the other hand, the main effect of the color of FLS on ERS was not significant at both Pz (*p* = 0.955, ηp^2^ < 0.001, Fig. 3C) and Fz (*p* = 0.261, ηp^2^ = 0.037, Fig. 3D). The main effect of the flickering frequency of FLS on ERS was significant at both Pz (F_4, 136_ = 9.584, *p* < 0.001, ηp^2^ = 0.220, Fig. 3E) and Fz (F_4, 136_ = 17.453 *p* < 0.001, ηp^2^ = 0.339, Fig. 3F). At both Pz and Fz, fundamental and harmonic responses became stronger as the flickering frequency of FLS decreased from 40 to 32 Hz. In the post hoc comparisons, 32 Hz or 34 Hz FLSs entrained stronger ERS than 38 Hz or 40 Hz at both Pz (*p* < 0.05) and Fz (*p* < 0.01). The interaction of the flickering frequency of FLS with the luminance intensity of FLS was not significant at both Pz (*p* = 0.781, ηp^2^ = 0.012) and Fz (*p* = 0.907, ηp^2^ = 0.007), while that with the color of FLS was statistically significant at Pz (F_4, 136_ = 3.383, *p* = 0.028, ηp^2^ = 0.090). Under red FLS, ERS entrained by 32 Hz FLS was stronger than those entrained by 36 Hz or higher (*p* < 0.05). However, under white FLS, ERS was comparably entrained at all flickering frequencies (*p* > 0.05).

### Effects of luminance intensity and color of FLS on the propagation of entrained gamma rhythms

**Figure 4:**
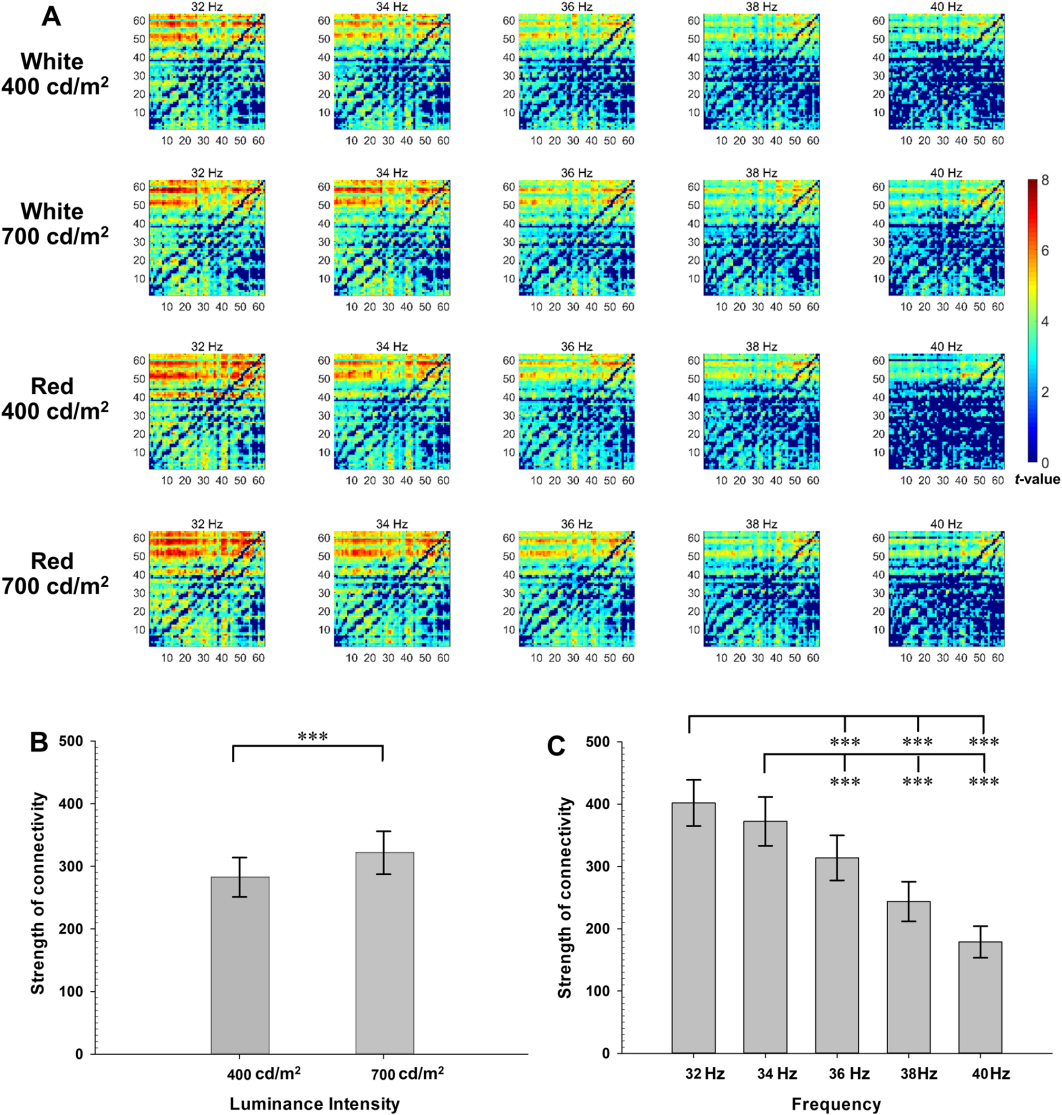
Changes in the gamma rhythm connectivity after flickering light stimulation. The spectral Granger causality (sGC) is analyzed in 1953 connections between 63 electrodes. The numbers electrodes from 1 to 63 correspond to Fp1, Fp2, AF7, AF3, AFz, AF4, AF8, F7, F5, F3, F1, Fz, F2, F4, F6, F8, FT9, FT7, FC5, FC3, FC1, FC2, FC4, FC6, FT8, FT10, T7, C5, C3, C1, Cz, C2, C4, C6, T8, TP9, TP7, CP5, CP3, CP1, CPz, CP2, CP4, CP6, TP8, TP10, P7, P5, P3, P1, P2, P4, P6, P8, PO7, PO3, POz, PO4, PO8, O1, Oz, and O2, respectively. (**A**) t-values in the spectral Granger causality of the occipitoparietal to frontotemporal gamma rhythm connections. The left-upper side of each matrix represents the occipitoparietal to frontotemporal connections. (**B**) Main effect of luminance intensity on the averaged strength of the occipitoparietal to frontotemporal gamma rhythm connections; (**C**) Main effect of flickering frequency on the averaged strength of the occipitoparietal to frontotemporal gamma rhythm connections. Error bars indicate standard errors. ^***^p < 0.001 by repeated-measures analysis of variance.

As shown in Fig. 4A, the connectivity of gamma rhythm increases significantly after FLSs in both parieto-occipital-to-frontotemporal and frontotemporal-to-parieto-occipital connection edges (*p* < 0.05, Cohen’s *d* illustrated in Supplementary Fig. 1). The number of edges in which parieto-occipital-to-frontotemporal connectivity^24,25^ of gamma rhythm increased after FLSs ranged from 1137 (58.2% within 1953 edges) after 400 cd/m^2^ red FLS of 40 Hz to 1769 (90.6% within 1953 edges) after 700 cd/m^2^ white FLS of 34 Hz. The main effect of the luminance intensity and flickering frequency of FLS on the strength of the parieto-occipital-to-frontotemporal connectivity were significant (F_1, 34_ = 15.903, *p* < 0.001, ηp^2^ = 0.319 for luminance intensity; Fig. 4B; F_4, 136_ = 58.469, *p* < 0.001, ηp^2^ = 0.632 for luminance frequency; Fig. 4C). The connectivity entrained by 700 cd/m^2^ FLS was stronger than that entrained by 400 cd/m^2^ FLS (*p* < 0.001). The connectivity entrained by 32 Hz FLS or 34 Hz FLS was also stronger than that entrained by the FLS of 36 Hz or higher (*p* < 0.001). However, the main effect of the color of FLS on the strength of connectivity was not significant (F_1, 34_ = 0.163, *p* = 0.689, ηp^2^ = 0.005). All the interactions between the luminance intensity, color, and flickering frequency of FLS on the strength of connectivity were not significant (*p* > 0.147, ηp^2^ = 0.030).

### Age associated decrease in the optimal frequency of FLS for gamma entrainment

When we compared the optimal FLS frequency between the young-old participants aged under 70 (12 males and 10 females; age, 67.9 ± 1.0 years) and the old-old participants aged 70 years or older (five males and eight females; 71.6 ± 1.8 years), the flickering frequency that entrained the strongest gamma rhythms in the young-old participants was higher than that in old-old participants (33.7 ± 2.8 Hz versus 32.2 ± 0.6 Hz; t = 2.569, *p* = 0.017, Cohen’s *d* = 0.533). However, the luminance and color that entrained the strongest gamma rhythms were not significantly different between young-old and old-old participants (U = 118.5, *p* = 0.408, *r* = 0.204 for luminance intensity; U = 146.5, *p* = 0.906,* r* = 0.024 for color).

### Adverse effect of FLS

All adverse effects were mild, and their severities were not significantly different between the four combinations of color and luminance (Table 1).

**Table 1:** Self-reported adverse effects of flickering light stimulation.

	White and 400 cd/m^2^	White and 700 cd/m^2^	Red and 400 cd/m^2^	Red and 700 cd/m^2^	F*	P*	ηp^2*^
Fatigue	1.3 (2.0)	1.3 (1.8)	1.0 (1.7)	1.0 (1.8)	0.01	1.000	0.000
Headache	0.4 (1.2)	0.5 (1.2)	0.4 (1.2)	0.3 (1.2)	0.09	0.761	0.003
Dizziness	1.1 (1.8)	1.1 (1.6)	1.1 (1.8)	0.9 (1.6)	0.13	0.726	0.004
Dazzling	2.0 (1.8)	2.1 (2.0)	2.0 (2.0)	2.2 (1.9)	0.57	0.454	0.017
Asthenopia	1.6 (1.8)	1.3 (1.9)	1.4 (1.6)	1.6 (1.9)	2.96	0.094	0.080
Ocular pain	0.1 (0.7)	0.0 (0.2)	0.1 (0.3)	1.1 (0.7)	0.67	0.419	0.019

*repeated measures analysis of variance.

## Discussion

In the current study, FLS of 700 cd/m^2^ entrained stronger and more widely spread gamma rhythm than that of 400 cd/m^2^ but showed no significantly different adverse effects to that of 400 cd/m^2^. In addition, FLSs of 32 Hz or 34 Hz entrained the stronger and more widely spread gamma rhythm than that of other higher flickering frequencies. However, white and red FLSs comparably entrained gamma rhythm in the brains of older adults.

In our previous study on younger adults, 700 cd/m^2^ FLS entrained stronger and more widely spread gamma rhythm than FLSs with low luminance intensities (400 cd/m^2^, 100 cd/m^2^, or 10 cd/m^2^), which was the first study on the effect of luminance intensity of FLS on the power and propagation of entrained gamma rhythm simultaneously in younger adults^19^. In line with our previous study, Jones et al. found that 377 cd/m^2^ FLS entrained stronger gamma rhythm at both occipital and frontal electrodes than 192 cd/m^2^ FLS in three healthy volunteers^16^. The current study clearly demonstrated that stronger FLS could entrain gamma rhythm more strongly and widely in older adults too. Compared with the ERS entrained by 400 cd/m^2^ FLS, that entrained by 700 cd/m^2^ FLS was 1.07 times and 1.03 times stronger at Pz and Fz, respectively. In addition, ERS entrained by 700 cd/m^2^ FLS was spread to 1.05 times more nodes from the parieto-occipital region to frontotemporal region with 1.14 times stronger connections than that entrained by 400 cd/m^2^ FLS. Although it is not fully understood yet how stronger light entrains stronger SSVEP, higher-amplitude light energy may induce more changes in the electrochemical properties of retinal photoreceptors and nerve conduction of visual pathways^26^.

FLS with longer wavelengths induced stronger SSVEP than FLS with shorter wavelengths in humans^17,27^. Retinal cones are responsible for color vision, and long-wavelength sensitive cones are denser than medium and short-wavelength sensitive cones in human^28,29^. Red FLS mostly excites medium-wavelength-cones (M-cones) and long-wavelength-cones (L-cones) while white FLS also excites short-wavelength-cones (S-cones) (Supplementary Table 1). Other studies have shown that excitation of L-cones strongly induced gamma rhythm in visual cortex^30^, whereas excitation of S-cone by light with shorter wavelength peak could not^31,32^. In general, longer wavelengths seem to be a better stimulus for gamma entrainment. This was also hinted in our prior study on young adults as red FLS showed a trend of stronger entertainment compared to the white FLS^19^. However, the difference was not significant, and in addition, study on older adults has shown no difference between entrainment of white and red FLS. We hypothesize that there are two reasons for the lack of difference in FLS color effect in older adults. Spectral irradiance of the white FLS used in this study mostly consists of the longer wavelength (Supplementary Fig. 3), and the increased senescence of lenses diminishes the transmittance for shorter wavelengths^33,34^ as shown in the Supplementary Fig. 10, which would diminish the shorter wavelengths in white FLS. These reasons may have reflected on our result by reducing the difference in the effect of red and white FLS on gamma entrainment. Therefore, in older adults, white (highest peak at 612 nm) color that is peaked at a longer wavelength can be as effective as the red (peak at 614 nm) color FLS for gamma entrainment.

Furthermore, the frequency and severity of adverse effects were mild and was not significantly different between 700 cd/m^2^ FLS and 400 cd/m^2^ FLS in older adults, which is important in clinical application of FLS. In a previous study on 10 patients with AD, adverse effects of approximately 700 cd/m^2^ FLS were also mild^35^. However, in our previous study on younger adults, adverse effects were more common and severe in 700 cd/m^2^ FLS compared with those in 400 cd/m^2^ FLS^19^. Older adults may be more tolerable to stronger light than younger adults, possibly due to an age-associated increase of miosis and lenticular senescence^36,37^. For the same reason, older adults may need stronger FLS to entrain as strong a gamma rhythm as younger adults. In addition, further studies are warranted to investigate the strongest luminance of FLS that can entrain gamma rhythm better than 700 cd/m^2^ without increasing adverse effects in older adults.

The power of entrained gamma rhythm may be related to the excitatory-inhibitory regulation of gamma-aminobutyric acid (GABA)-ergic inhibitory interneurons. It increases as the motion velocity of visual input increases^38,39^ because faster visual input increases the tonic excitability of GABAergic inhibitory interneurons^40,41^. The initial increase in the entrained gamma power during the transition from the static to slow-motion velocity may be attributable to the recruitment of a large fraction of excitatory and inhibitory neurons in synchronous activity during the transition from suboptimal to optimal input intensity^42,43^. However, stronger excitation within both excitatory and inhibitory circuitry, triggered by a further increase in the motion velocity, suppresses gamma synchrony^44^. Therefore, the power of entrained gamma rhythm may increase as the flickering frequency of FLSs increases to a certain level, but it will decrease afterward. This study found that optimal flickering frequencies for entraining gamma rhythm in older adults were a bit lower than those in younger adults. The flickering frequency that entrained the strongest and most widely spread gamma rhythm was 32 Hz or 34 Hz in the older adults in the current study, while it was 36 Hz or 38 Hz in younger adults in our previous study^19^. In older adults, 32 Hz or 34 Hz FLSs entrained gamma rhythms approximately 1.2 times stronger at Pz and 1.4 times stronger at Fz than 40 Hz FLS. In addition, 32 Hz and 34 Hz FLSs entrained gamma rhythms at approximately 1.3 times more nodes with approximately two times higher strength than 40 Hz FLS. However, in our previous study on young adults, 32 Hz FLS comparably entrained gamma rhythm to 40 Hz FLS. Instead, 38 Hz FLS entrained gamma rhythms approximately 1.2 times stronger at both Pz and Fz and at 2.1 times more nodes with 2.8 times higher strength than 40 Hz FLS. These results indicate that optimal flickering frequency for entraining gamma rhythm may decrease with advancing age in human. Even within older adults, the optimal flickering frequency for entraining gamma rhythm of the old-old participants was found to be approximately 1.5 Hz lower than that of the young-old participants.

This age-associated decrease in the optimal flickering frequency for gamma entrainment may be attributable to the age-associated decrease in the center frequency of gamma rhythms in humans. Gamma rhythms, generated in mutually connected GABAergic inhibitory interneuron networks, regulate global excitatory-inhibitory balance in the visual cortex^45–47^. Center frequency is the frequency where the power changes most in response to external visual stimulation^21,48^. It increases monotonically with increasing intensity of visual input such as visual contrast^42,49–51^and motion velocity^38,39^. It is positively correlated with GABA level in the visual cortex^52,53^ and increases as the tonic excitability of GABAergic inhibitory interneurons increases^40,41^. According to Murty et al., center frequency gradually decreased with advancing age (0.16 Hz per year in high gamma range of 36 Hz or higher and 0.08 Hz per year in low gamma range below 36 Hz)^21^. If we apply these results to the older participants of the current study (69.9 ± 2.3 years) and the younger participants of our previous study (24.1 ± 3.6 years), the differences of center frequency in high and low gamma bands are estimated to be 7.4 Hz and 3.7 Hz respectively between them. If we apply these results to the young-old participants (67.9 ± 1.0 years) and the old-old participants (71.6 ± 1.8 years) in the current study, the differences of center frequency in high and low gamma bands are estimated to be 0.5 Hz and 0.2 Hz respectively between them. This age-associated decrease in center frequency may be attributable to the age-associated decrease in the excitability of GABAergic inhibitory interneurons^54,55^. The contrast sensitivity to medium and high contrast and spatial frequencies decreases with advancing age^56^. Lower contrast sensitivity may induce less cortical excitation and consequently lower center frequency in the old population^57^. In addition, the level of GABA decreased in visual, sensory motor, frontal, and prefrontal cortices areas with advancing age in humans^58,59^, which may degrade inhibitory intracortical circuits^60–63^.

This study has several limitations to be noted. First, the participants were healthy volunteers. Optimal luminance or flickering frequency of FLS in patients with AD may be different from those in healthy older adults. Patients with AD showed smaller pupillary diameter than healthy older controls^64,65^. In addition, Aβ microaggregates in the lens may induce fluctuation of refractive index and increase light scattering^66,67^. Second, optimal luminance and flickering frequency for entraining gamma rhythm might be different between individuals because the center frequency of gamma rhythm^21,68^ and the degree of miosis^69^ and lens senescence^70^ was different between individuals. Third, the effects of FLS on cognitive performance or cerebral amyloid deposition were not examined. Fourth, the sample size was small and subject to limited statistical power.

Despite these limitations, the current study clearly demonstrate that gamma rhythm could be entrained widely in the brains of older adults by visual stimulation without additional stimulation of different sensory modalities if its parameters were optimized. In previous studies on AD mouse models, visual stimulation entrained gamma rhythm in higher-order brain areas, such as hippocampus and prefrontal cortex^12^, and expanded the regions where gamma rhythm was entrained by auditory stimulation^11^. Furthermore, visual stimulation reduced AD pathologies in the brain regions where gamma rhythm was entrained and improved cognitive function of mice in both studies^11,12^. Unimodal sensory stimulation using FLS can be a promising candidate for therapeutic treatment of AD, which is easier to apply and more cost-effective than multi-modal sensory stimulation.

## Methods

### Participants

We enrolled 46 cognitively normal volunteers who did not have psychiatric or neurologic disorders and were aged 60 years or older (22 men and 24 women; age, 69.9 ± 2.3 years). Geriatric psychiatrists evaluated participants through face-to-face standardized diagnostic interviews, physical and neurologic examinations, and laboratory tests using the Korean version of the Consortium to Establish a Registry for Alzheimer’s Disease Assessment Packet^71^ and the Korean version of the Mini International Neuropsychiatric Interview^72^. All participants had normal or corrected-to-normal vision and normal hearing. None of them had a current or previous history of major psychiatric or neurological disorders, including epilepsy. Finally, 35 participants (17 men and 18 women; age, 70.0 ± 2.4 years) were included in the final analysis after excluding 11 participants for the following reasons: three participants withdrew their consents during experiments, and eight had excessive electromyogram (EMG) noises in their electroencephalogram (EEG). The mean scores of the Mini Mental Status Examination and the Geriatric Depression Scale were 28.17 ± 2.05 and 7.06 ± 4.97, respectively. All participants provided written informed consent. This study was approved by the institutional review board (IRB) of Seoul National University Bundang Hospital (IRB No.: B-1809-493-004), and was performed in accordance with relevant guidelines and regulations.

### Flickering light stimulation

FLS was delivered using a pair of white organic light-emitting diode (OLED) panels (4.7 cm × 4.7 cm; color temperature 3000 K; LG Display Co., Ltd., Seoul, Korea) attached to an eyeglass. Voltage-luminance characteristics of OLED panels were measured using a calibrated spectroradiometer (CS2000, Konica-Minolta Inc. Tokyo, Japan) at voltage-controlled mode using a precise source measurement unit (Keithley 2400, Tektronix Inc., Beaverton, OR, USA). The OLED panels were driven with a square rhythm using a function generator (TG 5012A, Aim & Thurlby Thandar Instruments, Huntingdon, Cambridgeshire, UK) with 100% modulation depth and 50% duty cycle. The error of voltage fluctuation was controlled under 5 mV, and the corresponding error of luminance was estimated to be less than 5%. The amplitude and frequency of the square rhythm were modulated to change the luminance and frequency of FLS using an in-house LabVIEW program (National Instruments Corporation, Austin, TX, USA). The red color of FLS was realized by placing an optical color filter (KODAK WRATTEN 2, Eastman Kodak Company; No. 25 for red) in front of the white OLED. Red OLEDs had the spectral emissions ranging from ca. 590 nm to 700 nm with the peak at the wavelength of 614 nm and the full width half maximum of 54 nm. White OLEDs had the spectral emission ranging from ca. 440 nm to 700 nm with the three major peaks at the wavelengths of 454 nm, 540 nm, and 612 nm, of which the peak at 612 nm exhibited the highest value. We provided spectral power distributions of all the 4 stimuli (white 400 cd/m^2^, white 700 cd/m^2^, red 400 cd/m^2^, and red 700 cd/m^2^) and excitations of the five photoreceptor classes (S-cones, M-cones, L-cones, rod, and Intrinsically photosensitive retinal ganglion cells) for each stimulus (Supplementary Figs. 2–7 and Table 1). The luminance of FLS was controlled by changing the supply voltage of the OLED for each color. For 400 cd/m^2^ and 700 cd/m^2^ of red FLS, 8.31 V and 8.72 V were supplied, respectively. In addition, for 400 cd/m^2^ and 700 cd/m^2^ of white FLS, 7.71 V and 7.91 V were supplied (Supplementary Figs. 8 and 9). For all colors and luminance values, FLS was provided at distinctive five flickering frequencies (32 Hz, 34 Hz, 36 Hz, 38 Hz, and 40 Hz) in the gamma-rhythm range based on our previous study^19^, which examined more effective frequencies of FLS to entrain gamma EEG in healthy young adults. The distance between the OLED panel and the cornea was set to 2 cm. The subtended angles seen by the center point of the cornea for the middle points of edges and the vertex point of the OLED panel were 99.1 and 118.0 degrees, respectively.

### Experimental design

**Figure 5:**
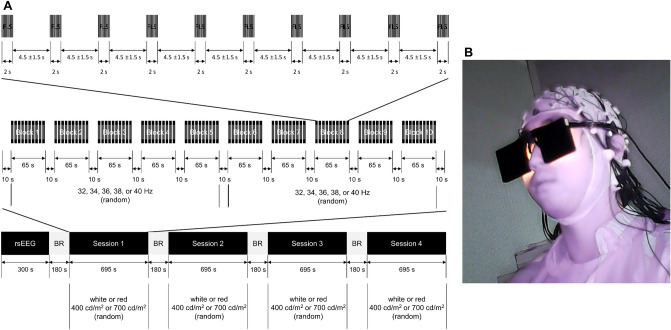
(**A**) Experimental procedures FLS, flickering light stimulation; rsEEG, resting-state electroencephalogram; BR, break (**B**) An example of a subject wearing a flickering light stimulation device.

All participants were instructed to fast for at least 2 h and not to drink alcohol 24 h before the experiment. This study consisted of a 5 min resting phase for recording resting-state EEG (rsEEG) and four experimental sessions for recording SSVEPs (Fig. 5A). To control the potential confounding effect of the experimental design, we randomized the order of FLS parameters to prevent habituation and set the interval between sessions as three minutes which is long enough to wash out the effect of prior session. All intervals between the resting phase and the four experimental sessions were 3 min. One of four light sources (400 cd/m^2^ white light, 700 cd/m^2^ white light, 400 cd/m^2^ red light, and 700 cd/m^2^ red light) was assigned to each session. A session consisted of 10 blocks. A 10 s break was placed before and after each block. Each block consisted of 10 FLS trials of a given luminance intensity, color, and flickering frequency. One of the five flickering frequencies (32 Hz, 34 Hz, 36 Hz, 38 Hz, and 40 Hz) was randomly assigned to each block. Since each session consisted of 10 blocks, each flickering frequency was assigned to two blocks in each session. Each FLS was presented for 2 s, and the inter-FLS interval was randomly given from 3 to 6 s (4.5 ± 1.5 s).

rsEEG was recorded for 5 min while the participants stayed still with their eyes closed. In each session, EEG was recorded for 11 min and 55 s while the participants stayed still with their eyes open with a flickering light stimulus device (Fig. 5B). We obtained an informed consent to use the picture (Fig. 5B) from the volunteer for the online open-access publication. After each session, the participants were asked to rate the severity of six adverse effects of FLS (fatigue, headache, dizziness, dazzling, asthenopia, and ocular pain) using a 7-point Likert-type scale from 0 (not at all) to 6 (extremely severe).

### Electroencephalography recording and preprocessing

EEG was recorded using 64 Ag–AgCl electrodes attached to an elastic cap (Easycap, EASYCAP GmbH, Munich, Germany) according to the extended International 10–20 System. The reference electrode was FCz. The ground electrode was placed on the forehead, and a pair of electrodes was additionally attached below and above the left eye to record an EMG. All electrodes were maintained at an impedance of 10 kΩ or less during the entire recording. The recorded EEG signal was amplified and stored using a 24-bit ActiCHamp DC amplifier and BrainVision Recorder (Brain Products Gmbh, Gliching, Germany). The sampling rate was set at 1000 Hz. Online filters were not applied during EEG recording. The stimulus markers were delivered from the FLS control system and were synchronized with the recording.

All preprocessing and analysis procedures were conducted using MATLAB (The MathWorks Inc., Natick, MA, USA), EEGLAB^73^, and BSMART^74^ toolbox. The recorded signals were filtered with a 1 Hz high-pass finite impulse response filter and a 60 Hz notch filter and then applied to a common average reference. An independent component analysis was performed to remove eye blinks and other ocular artifacts from the EEG signal.

After preprocessing, 5 min rsEEG recordings were segmented into 1500 ms epochs, and then 20 artifact-free epochs were randomly selected from them. A 4000 ms epoch was obtained from 1000 ms before each FLS onset to 1000 ms after each FLS offset, resulting in 20 4000 ms epochs of each frequency. For the spectral Granger causality (sGC) analysis, 1500 ms was obtained from 501 ms to 2000 ms from each 4000 ms epoch.

### Analyses

To find the optimal color, luminance, and frequency of FLS for entraining gamma rhythms in aging, we analyzed the spectral power change of EEG induced by FLS using event-related spectral perturbation (ERSP) in each block. We calculated the time-frequency spectrum in each epoch and normalized the spectrum by dividing the average power of pre-stimulus intervals. We calculated the ratio of the spectral power of EEG during FLS to that during the pre-FLS interval for each FLS. To get a normalized averaged spectral power change induced by a given color, luminance, and frequency of FLS, we calculated the ERD/ERS value by averaging the ratios of the spectral power of EEG from 20 FLS trials of a given FLS frequency in a session. We also calculated the time-associated change of power spectrum by averaging ERSP of 11 successive, non-overlapping, 250 ms subwindows from 250 ms before FLS onset to 2500 ms after FLS onset in each block (T0–T10). T0 (− 250–0 ms) represents the time windows right before FLS onset, and T9 (2000–2250 ms) and T10 (2250–2500 ms) represent the time windows right after FLS offset. Then, we compared ERS between these 11 blocks (T0–T10) to confirm that FLS entrained gamma rhythm using repeated measures analysis of variance (rmANOVA) with the Greenhouse–Geisser non-sphericity correction and Bonferroni post hoc comparisons. We used the average ERS during FLS (T1–T8) in the analyses on the effects of luminance intensity, color, and flickering frequency on gamma entrainment. We selected Pz in the parieto-occipital region because SSVEP of visual stimuli was observed dominantly at Pz in previous studies^75,76^. We selected Fz in addition to prove that gamma rhythms entrained in the occipital lobe spread well throughout the brain because Fz is one of the leads that are most apart from the occipital region. With 2 (luminance: 400 cd/m^2^, 700 cd/m^2^) × 2 (color: white, red) × 5 (flickering frequency: 32 Hz, 34 Hz, 36 Hz, 38 Hz, and 40 Hz) design rmANOVA, we examined the effect of luminance, color, and flickering frequency of FLS on the changes of gamma rhythm associated with FLS with the Greenhouse–Geisser non-sphericity correction and Bonferroni post hoc comparisons. We also compared the optimal FLS frequency using Student t-test and the optimal FLS luminance (400 cd/m^2^, 700 cd/m^2^) and color (white, red) using Mann–Whitney U tests between the young-old group (12 males and 10 females; age, 67.9 ± 1.0 years) and the old-old group (five males and eight females; 71.6 ± 1.8 years)^77–79^.

To examine whether gamma rhythms entrained in the occipital cortex propagate to other brain areas, we compared the sGC of gamma rhythms during FLS to that in the resting state. We calculated sGC for all possible electrode pairs and averaged each FLS frequency in each session. We set the time lags to 75 samples for calculating sGC^80^. We compared the mean sGC of the 20 1500 ms epochs from rsEEG with that of 20 1500 ms epochs from SSVEP during FLS using paired *t* test with FDR corrected *p* value of 0.05. Since EEG of each FLS condition had different pairs of significant connections between electrodes, we employed graph theory measures to compare the network structures quantitatively between conditions of luminance intensity, color, and flickering frequency^81,82^. We constructed an adjacency matrix of a given luminance, color, and flickering frequency of FLS using the edges that were found to be significantly different between rsEEG and SSVEP using paired *t *tests. We compared the number of edges where sGC of connection significantly increased after FLS between occipitoparietal to frontotemporal connection and frontotemporal to occipitoparietal connection. With 2 (luminance: 400 cd/m^2^, 700 cd/m^2^) × 2 (color: white, red) × 5 (flickering frequency: 32 Hz, 34 Hz, 36 Hz, 38 Hz, and 40 Hz) design rmANOVA, we examined the effects of luminance, color, and flickering frequency of FLS on the strength of connection during FLS with the Greenhouse–Geisser non-sphericity correction and Bonferroni post hoc comparisons.

Senescence of the lens may affect the sensitivity of the color FLS. Therefore, we investigated the effect of age on the irradiance of non-narrow band light sources from FLS source OLED panel (Supplementary Fig. 10).

With six measures of adverse effects (fatigue, headache, dizziness, dazzling, asthenopia, and ocular pain) and four levels of sessions (400 cd/m^2^ white light, 700 cd/m^2^ white light, 400 cd/m^2^ red light, and 700 cd/m^2^ red light) design rmANOVA, we examined the severity of adverse effects between 4 sessions of FLS with the Greenhouse–Geisser non-sphericity correction and Bonferroni post hoc comparisons.

## Supplementary Information

Supplementary Information.

## Data Availability

The data that support the findings of this study are available from the corresponding author upon reasonable request.
