# Supplementary material for: Optimal flickering light stimulation for entraining gamma rhythms in older adults
Source: Sci Rep. 2022 Sep 16;12:15550. doi: 10.1038/s41598-022-19464-2 (PMC9481621; doi:10.1038/s41598-022-19464-2)
Supplement: Supplementary file 1 — Supplementary Information. [file 41598_2022_19464_MOESM1_ESM.pdf]

# Optimal flickering light stimulation for entraining gamma rhythms in older adults

Yeseung Park[1,2,†] , Kanghee Lee[1,†], Jaehyeok Park[3], Jong Bin Bae[1,7], Sang-Su Kim[4], Do-Won Kim[4], Se Joon Woo [5,6], Seunghyup Yoo[3], Ki Woong Kim[1,2,7,*]

[1]Department of Neuropsychiatry, Seoul National University Bundang Hospital, Seongnam, Republic of Korea

[2]Department of Brain and Cognitive Science, Seoul National University, Seoul, Republic of Korea

[3]School of Electrical Engineering, Korea Advanced Institute of Science and Technology (KAIST), Daejeon, Republic of Korea

[4]Department of Biomedical Engineering, Chonnam National University, Yeosu, Republic of Korea

[5]Department of Ophthalmology, Seoul National University, College of Medicine, Seoul, Republic of Korea

[6]Department of Ophthalmology, Seoul National University Bundang Hospital, Seongnam, Republic of Korea

[7]Department of Psychiatry, Seoul National University, College of Medicine, Seoul, Republic of Korea

[†]These authors have contributed equally to this work and share first authorship

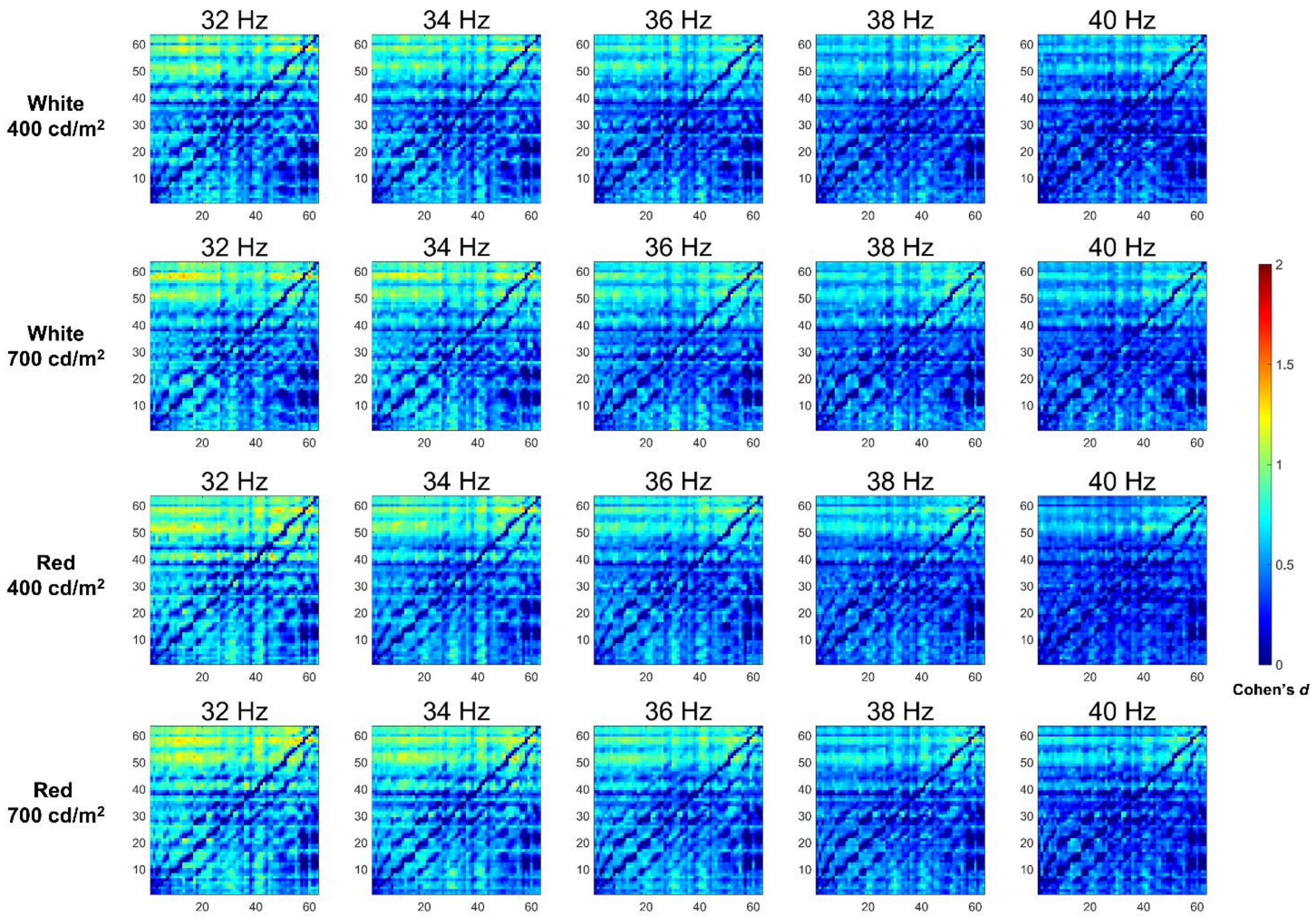


**Supplementary Figure 1**. Effect size of the gamma rhythm connectivity after flickering light stimulation. The spectral Granger causality is analyzed in 1,953 connections between 63 electrodes. The numbers electrodes from 1 to 63 correspond to Fp1, Fp2, AF7, AF3, AFz, AF4, AF8, F7, F5, F3, F1, Fz, F2, F4, F6, F8, FT9, FT7, FC5, FC3, FC1, FC2, FC4, FC6, FT8, FT10, T7, C5, C3, C1, Cz, C2, C4, C6, T8, TP9, TP7, CP5, CP3, CP1, CPz, CP2, CP4, CP6, TP8, TP10, P7, P5, P3, P1, P2, P4, P6, P8, PO7, PO3, POz, PO4, PO8, O1, Oz, and O2, respectively.

**Supplementary Method A**

**Distribution of spectral power in the range of wavelengths and the excitation of five photoreceptor classes by four light stimuli**

There are five photoreceptors in human vision system and each of them is activated by different wavelengths of light stimuli. The spectral sensitivity curves are standardized in CIE 026/E:2018 (**Supplementary Figure. 2**). In this study, the OLED light sources have broadband spectra and the light stimuli might activate all of the photoreceptors (**Supplementary Figure. 3**). To estimate the degree of excitation, the spectral overlap between the light source and each photoreceptor class was calculated using the following procedure.

1. Calculate $\bar{s}(\lambda)$, the spectral irradiance at cornea surface at the same irradiance, depends on the light sources.

2. Calculate $E_v$, the illuminance induced by the light sources with the same irradiance.

3. Calculate $E_e(\lambda)$, the spectral irradiance induced by light sources with the same luminance proportional to 400 or 700 cd/m$^2$ by dividing the spectral irradiance obtained in step 1 by the conversion factor obtained in the step 2.

4. Calculate $\hat{s}_{pr}(\lambda)$, the normalized spectral sensitivity of five photoreceptor classes for overlap integral.

5. Calculate $S$, the overlap integral between the values obtained in step 3 and step 4.

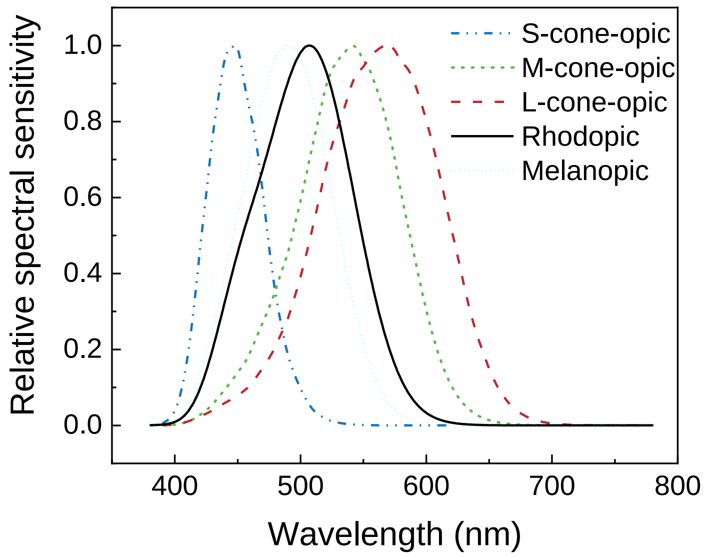


**Supplementary Figure 2.** Action spectra of five photoreceptors in human vision system. Data obtained from CIE S 026/E:2018.

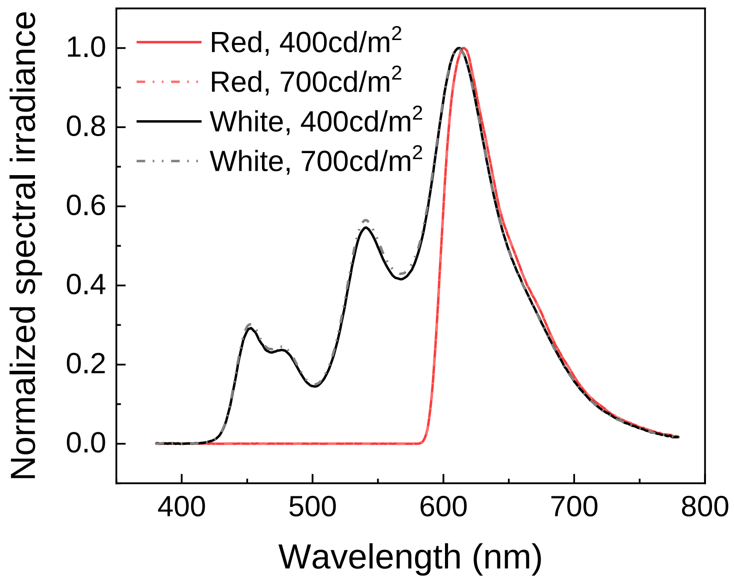


**Supplementary Figure 3.** Normalized spectral irradiance of white and red OLED light source used in the study.

Step 1. Calculate $\bar{s}(\lambda)$, the spectral irradiance at cornea surface with the same irradiance, depends on the light sources (**Supplementary Figure. 4**).

We arbitrarily choose irradiance of $1\text{W/m}^2$ as a reference value and calculated spectral irradiance of light sources satisfies the following condition (**Equation S1**). We set wavelength interval $\Delta\lambda$ equals to 1nm, following the convention used in the CIE 1931 color matching

$$\int_{\lambda} \bar{s}(\lambda)\,\mathrm{d}\lambda = \sum_{\lambda} \bar{s}(\lambda)\Delta\lambda = 1\text{W/m}^2 \qquad \textbf{(Equation S1)}$$

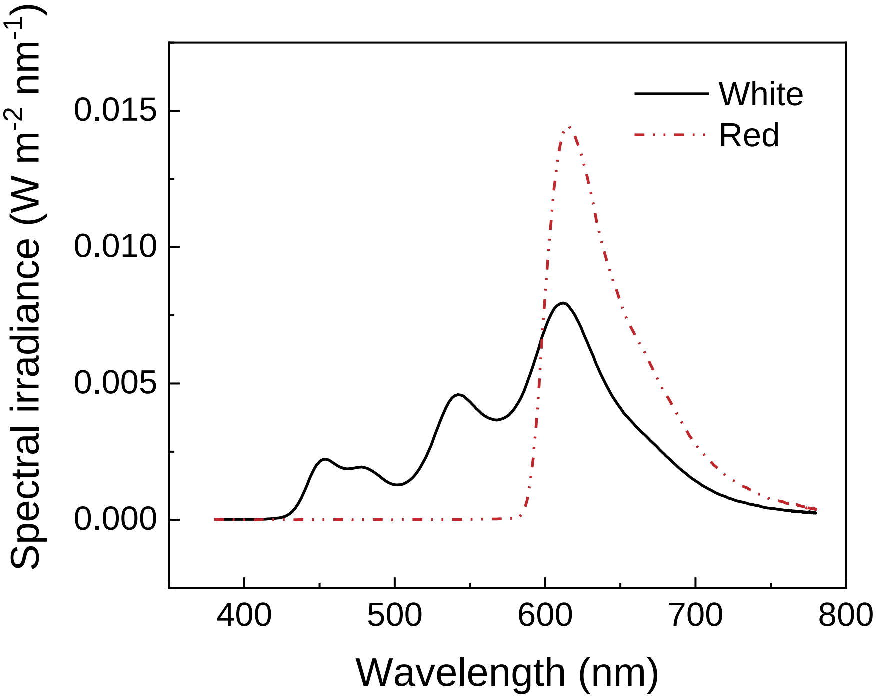


**Supplementary Figure. 4.** Calculated spectral irradiance at cornea surface with the irradiance of $1\text{W/m}^2$, depends on the light sources.

Step 2. Calculate $E_v$, the illuminance induced by the light sources with the same irradiance.

We take photopic response curve $\bar{y}(\lambda)$ defined in ISO/CIE 10527-1991 to convert irradiance to illuminance (**Supplementary Figure. 5**). The illuminance is obtained using following relations (**Equation S2**).

$$E_v = 683\int_{\lambda} \bar{s}(\lambda)\,\bar{y}(\lambda)\,\mathrm{d}\lambda = 683\sum_{\lambda} \bar{s}(\lambda)\,\bar{y}(\lambda)\Delta\lambda \qquad \textbf{(Equation S2)}$$

Resulting $E_v$ values on cornea surface induced by white and red OLED light sources with the same irradiance of $1\mathrm{W/m^2}$ are 308.83 and 173.10 $\mathrm{lm/m^2}$, respectively.

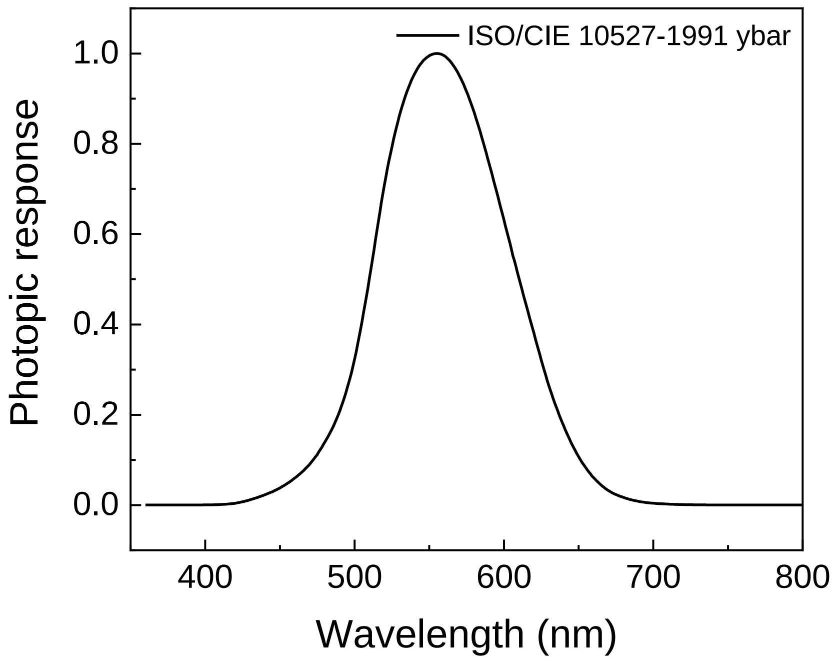


**Supplementary Figure 5.** Photopic response function of CIE 1931 Standard 2-degree Colorimetric observer (360-830nm)

Step 3. Calculate $E_e(\lambda)$, the spectral irradiance of light sources with the same luminance proportional to 400 or 700 cd/m$^2$ (**Supplementary Figure. 6**).

We divide the spectral irradiance obtained in step 1 by the conversion factor obtained in the step 2 (**Equation S3**).

$$E_{e,400}(\lambda) \propto \frac{1}{683}\frac{\bar{s}(\lambda)}{E_v}$$ (**Equation S3**)

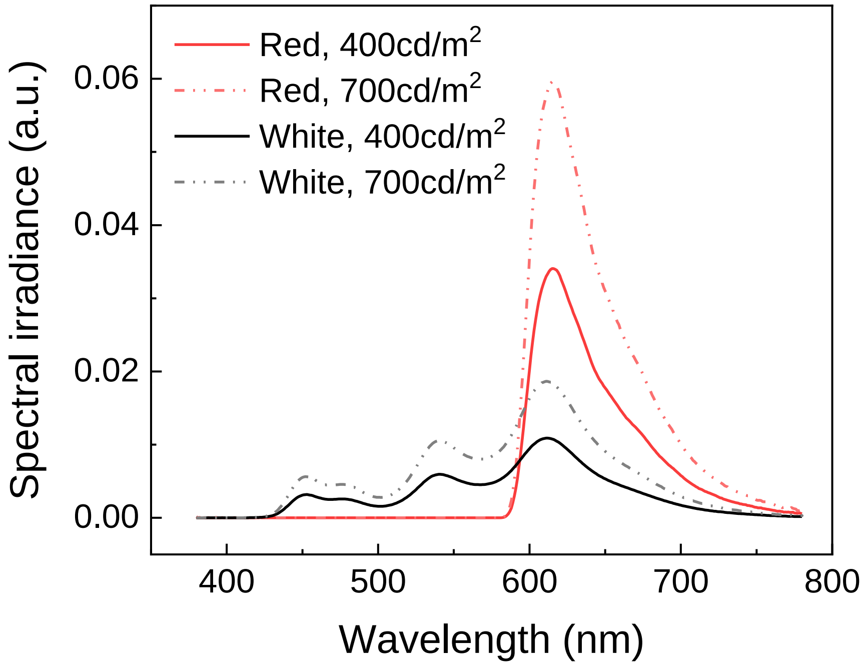


**Supplementary Figure 6.** Calculated spectral irradiance at cornea surface with the same luminance proportional to 400 or 700cd/m$^2$.

Step 4. Calculate $\hat{s}_{pr}(\lambda)$, the normalized spectral sensitivity of five photoreceptor classes for overlap integral (**Supplementary Figure. 7**).

Area normalized spectral sensitivity satisfies following relation (**Equation S4**).

$$\hat{s}_{pr}(\lambda) = s_{pr}(\lambda) / \int_{\lambda} s_{pr}(\lambda)\,d\lambda \qquad \textbf{(Equation S4)}$$

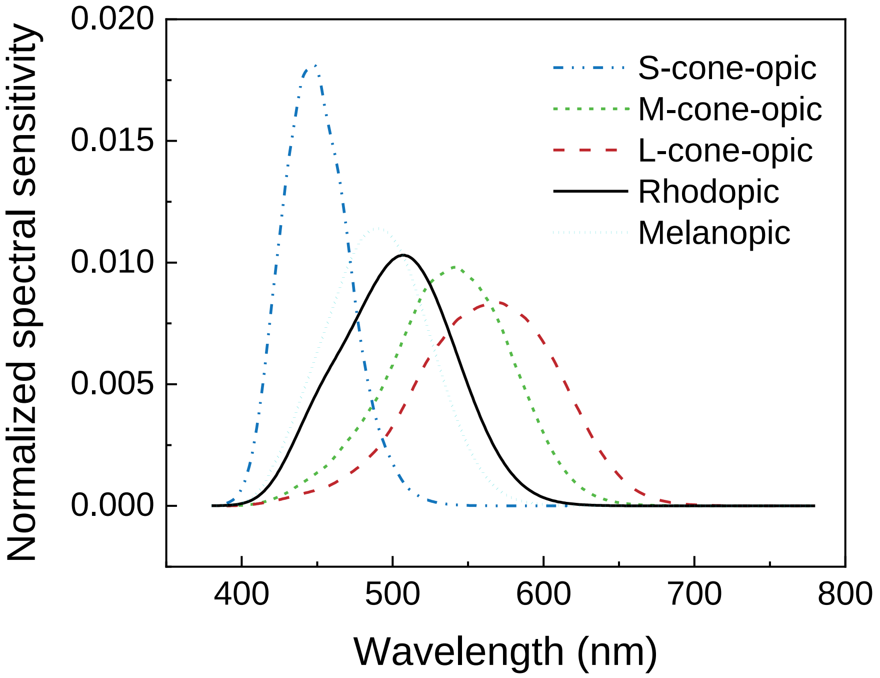


**Supplementary Figure 7.** Normalized spectral sensitivity of five photoreceptor classes for overlap integral.

Step 5. Calculate *S*, the overlap integral between the values obtained in step 3 and step 4.

Overlap integral S is obtained using following equations (**Equation S5**).

$$S = \int_{\lambda} \hat{s}_{pr}(\lambda) E_{e,400}(\lambda)\, d\lambda \equiv \sum_{\lambda} \hat{s}_{pr}(\lambda) E_{e,400}(\lambda)\, \Delta\lambda \qquad \textbf{(Equation S5)}$$

Resulting overlap integral for five photoreceptor classes are presented in **Supplementary Table 1**. We get single red/white ratio $r_{RW}$ using a following equation (**Equation S6**).

$$r_{RW} = S_{Red} / S_{White} \qquad \textbf{(Equation S6)}$$

**Supplementary Table 1.** Spectral overlap of five photoreceptor classes with the light stimuli

| | $L_e$=400cd/m$^2$ | | | $L_e$=700cd/m$^2$ | | |
|---|---|---|---|---|---|---|
| | Red | White | Red/white ratio | Red | White | Red/white ratio |
| S-cone-opic | $4.34\times10^{-6}$ | $1.86\times10^{-3}$ | 0.00 | $7.89\times10^{-6}$ | $3.31\times10^{-3}$ | 0.00 |
| M-cone-opic | $1.92\times10^{-3}$ | $4.43\times10^{-3}$ | 0.43 | $3.36\times10^{-3}$ | $7.80\times10^{-3}$ | 0.43 |
| L-cone-opic | $6.53\times10^{-3}$ | $5.57\times10^{-3}$ | 1.17 | $1.14\times10^{-2}$ | $9.73\times10^{-3}$ | 1.17 |
| Rhodopic | $1.86\times10^{-4}$ | $3.08\times10^{-3}$ | 0.06 | $3.26\times10^{-4}$ | $5.45\times10^{-3}$ | 0.06 |
| Melanopic | $3.28\times10^{-5}$ | $2.64\times10^{-3}$ | 0.01 | $5.79\times10^{-5}$ | $4.68\times10^{-3}$ | 0.01 |

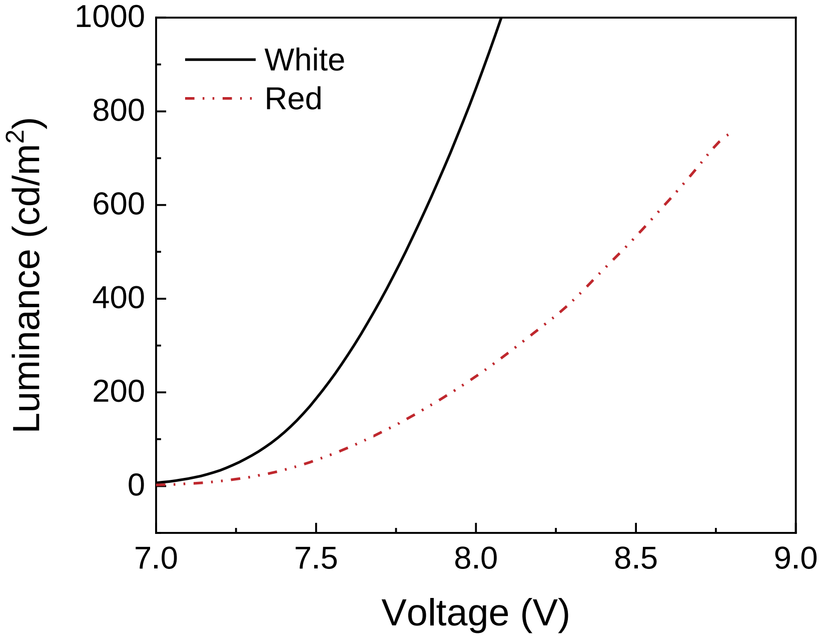


**Supplementary Figure 8.** Luminance versus voltage characteristics of white and red OLED light sources

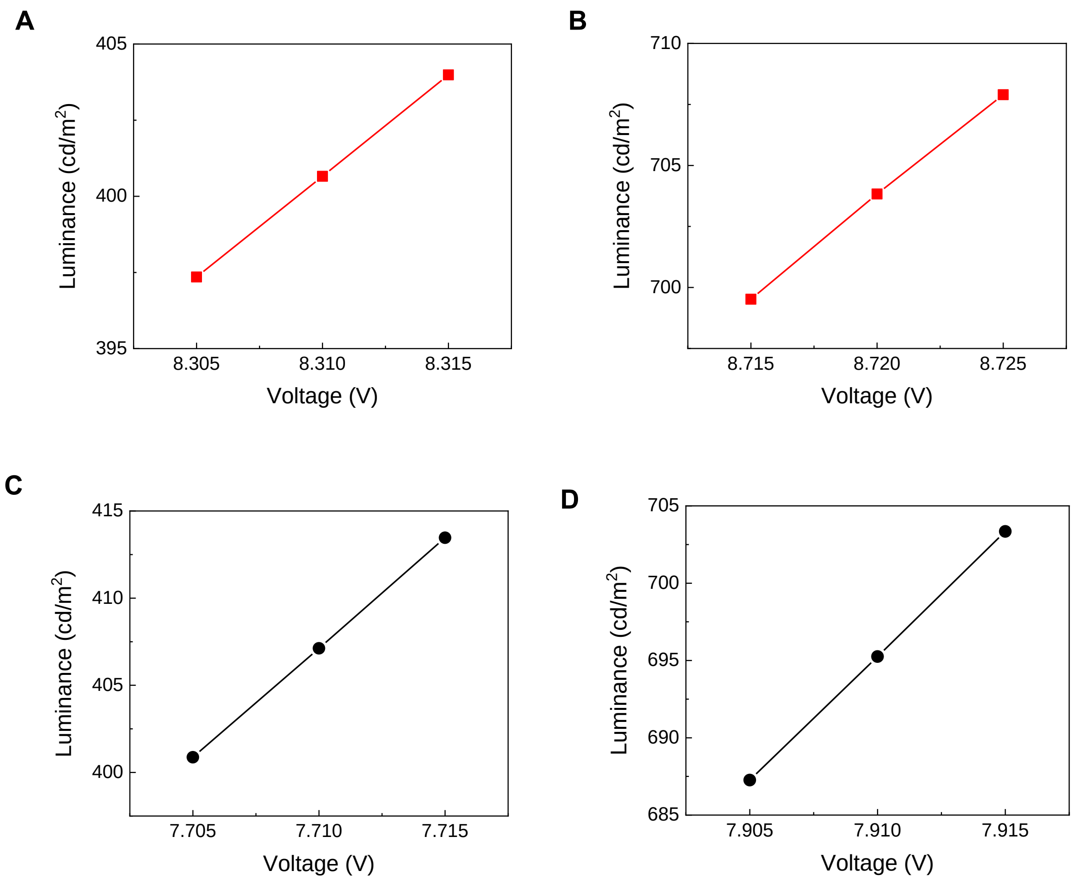


**Supplementary Figure 9.** Luminance versus voltage characteristics around the driving conditions for 400cd/m$^2$ and 700cd/m$^2$. (A) Luminance of red OLEDs with supply voltage near 8.31V. (B) Luminance of red OLEDs with supply voltage near 8.72V. (C) Luminance of white OLEDs with supply voltage near 7.71V. (D) Luminance of white OLEDs with supply voltage near 7.91V.

**Supplementary Method B**

**Characteristics of human ocular media in the visible and UV**

The optical density of human ocular media for age of 20 (Supplementary Figure. 10A) and 70 (Supplementary Figure. 10C) was calculated based on Equation S7[1] respectively. The transmittance of human ocular media for age of 20 (Supplementary Figure. 10B) and 70 (Supplementary Figure. 10D) was calculated using Equation S8, respectively.

$$\begin{aligned} D_{\mathrm{media}}(\lambda) = & (0.446 + 0.000031 \times age^2) \times (400/\lambda)^4 \\ & + 14.19 \times 10.68 \times e^{-(0.057*(\lambda-273))^2} \\ & + (0.998 - 0.000063 \times age^2) \times 2.13 \times e^{-(0.029*(\lambda-370))^2} \\ & + (0.059 + 0.000186 \times age^2) \times 11.95 \times e^{-(0.021*(\lambda-325))^2} \\ & + (0.016 + 0.000132 \times age^2) \times 1.43 \times e^{-(0.008*(\lambda-325))^2} + 0.225 \end{aligned}$$

**(Equation S7)**

$$\frac{I}{I_0} = 10^{-D}$$

**(Equation S8)**

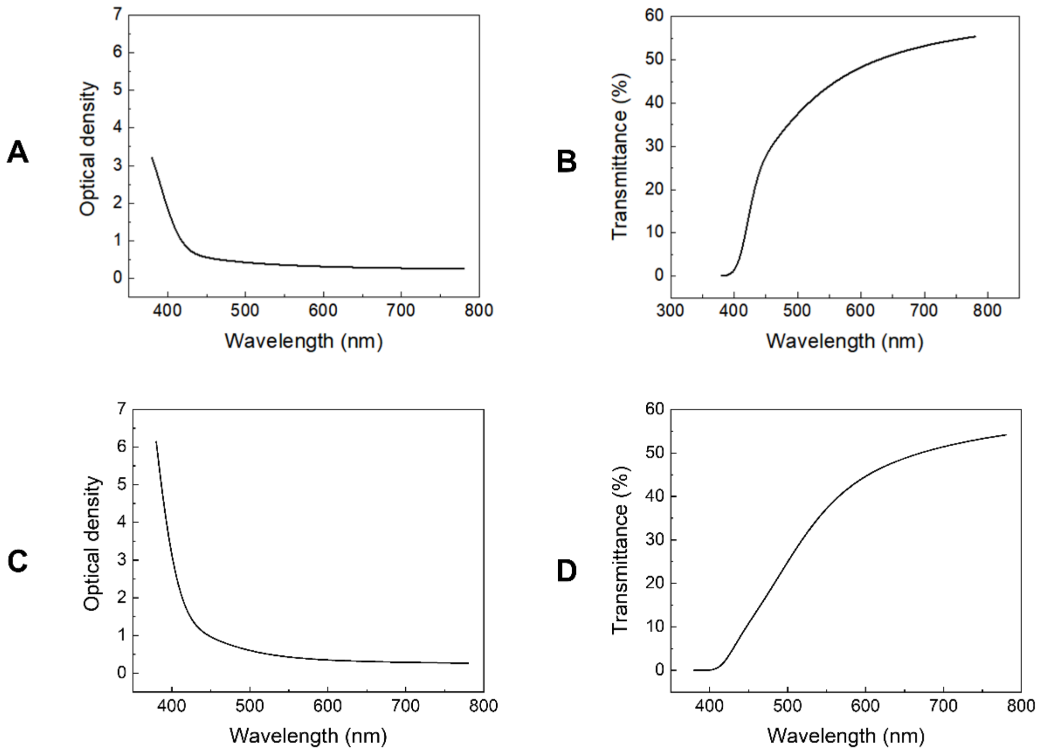


**Supplementary Figure 10.** Calculated characteristics of human ocular media for age of 20-year (A), (B) and 70-year (C), (D).

**References**

1. van de Kraats, J. & van Norren, D. Optical density of the aging human ocular media in the visible and the UV. *J. Opt. Soc. Am. A* **24**, 1842 (2007).
